# Inflammatory Myofibroblastic Tumor of the Lung: A Report of a Rare Case

**DOI:** 10.7759/cureus.63892

**Published:** 2024-07-05

**Authors:** Jayashree Bhawani, Samarth Shukla, Sourya Acharya

**Affiliations:** 1 Department of Pathology, Jawaharlal Nehru Medical College, Datta Meghe Institute of Higher Education and Research, Wardha, IND; 2 Department of Medicine, Jawaharlal Nehru Medical College, Datta Meghe Institute of Higher Education and Research, Wardha, IND

**Keywords:** alk-1, pulmonary neoplasm, imt, pseudotumor, pleural

## Abstract

The uncommon and mysterious pulmonary inflammatory myofibroblastic tumor (PIMT) primarily affects children and young people. PIMT is characterized by the proliferation of myofibroblastic spindle cells mixed with inflammatory cells. It can resemble both benign and malignant disorders, both radiographically and clinically. PIMT typically manifests as a solitary lung tumor. The genesis of the tumor is linked to genetic anomalies, including those related to the *ALK* gene (anaplastic lymphoma kinase); nonetheless, some cases are not ALK-positive, indicating genetic variability. Clinically, patients may have non-specific symptoms such as cough, chest pain, or hemoptysis, or they may not exhibit any symptoms at all. In these cases, imaging tests may unintentionally reveal unrelated conditions. From a histopathological perspective, PIMT is characterized by a heterogeneous cellular makeup, encompassing lymphocytes, myofibroblasts, plasma cells, and histiocytes, which generally exhibit a fascicular or storiform pattern. The diagnosis is verified using immunohistochemical labeling, molecular research, and histological examination. The cornerstone of treatment is still surgical resection, which has a good prognosis and a low recurrence rate. On the other hand, specific treatments, such as ALK inhibitors, have shown promise for incurable or recurring instances. Even though PIMT usually has a benign history, it is important to comprehend its biological behavior and molecular foundations for precise diagnosis and efficient management. This underscores the need for additional study into the pathophysiology and potential treatments of PIMT. This report presents a case of a 53-year-old female who presented with complaints of breathlessness and chest pain and was diagnosed with the condition accidentally.

## Introduction

The term "inflammatory pseudotumor" (IPT) describes a group of neoplastic and non-neoplastic entities with similar histological features, such as a prominent and usually persistent inflammatory infiltrate and spindle cell growth without cytology.

In the general category of inflammatory pseudotumors, the inflammatory myofibroblastic tumor (IMT) emerged in 1990, exhibiting unique clinical, pathological, and molecular characteristics [1]. Although Brunn initially reported it in 1939, its etiology is still unknown. IMTs are now categorized as uncommon intermediate-grade neoplasms with minimal metastatic potential and a high recurrence rate following excision [2,3].

IMTs account for 0.04% to 0.1% of all pulmonary neoplasms. They are more common in children and non-smoking adults [4,5]. Its clinical behavior is quite variable and is usually benign in nature. However, the possibility of metastasis cannot be denied in several cases. Diagnosis is often challenging and, many times, only possible after resection of the tumor. IMT is highly uncommon in adult females and rarely documented in the lungs. This study presents a highly uncommon case of a patient with pulmonary IMTs.

## Case presentation

A 53-year-old married female presented to our healthcare center in February 2024 with complaints of breathlessness accompanied by chest discomfort and pain, which she had been experiencing for 10 days. There were no apparent symptoms of an irritating dry cough or sputum throughout this period. She was given symptomatic treatment and was sent back, but her condition did not improve. She came to the respiratory medicine outpatient department (OPD) after three days, and radiological investigations were performed.

**Figure 1:**
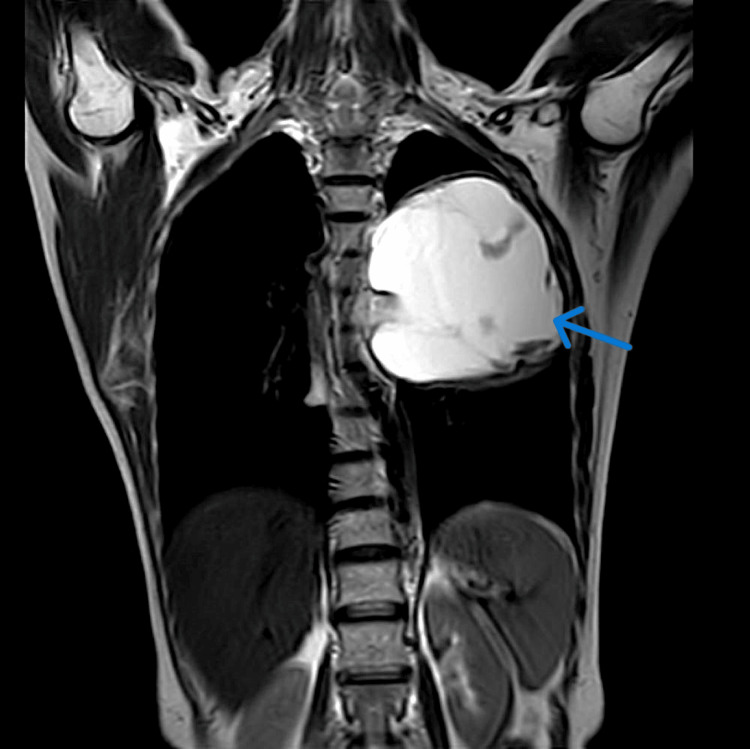
MRI image showing evidence of a well-defined T2 hyperintense lesion (blue arrow) in the upper lobe of the left lung.

**Figure 2:**
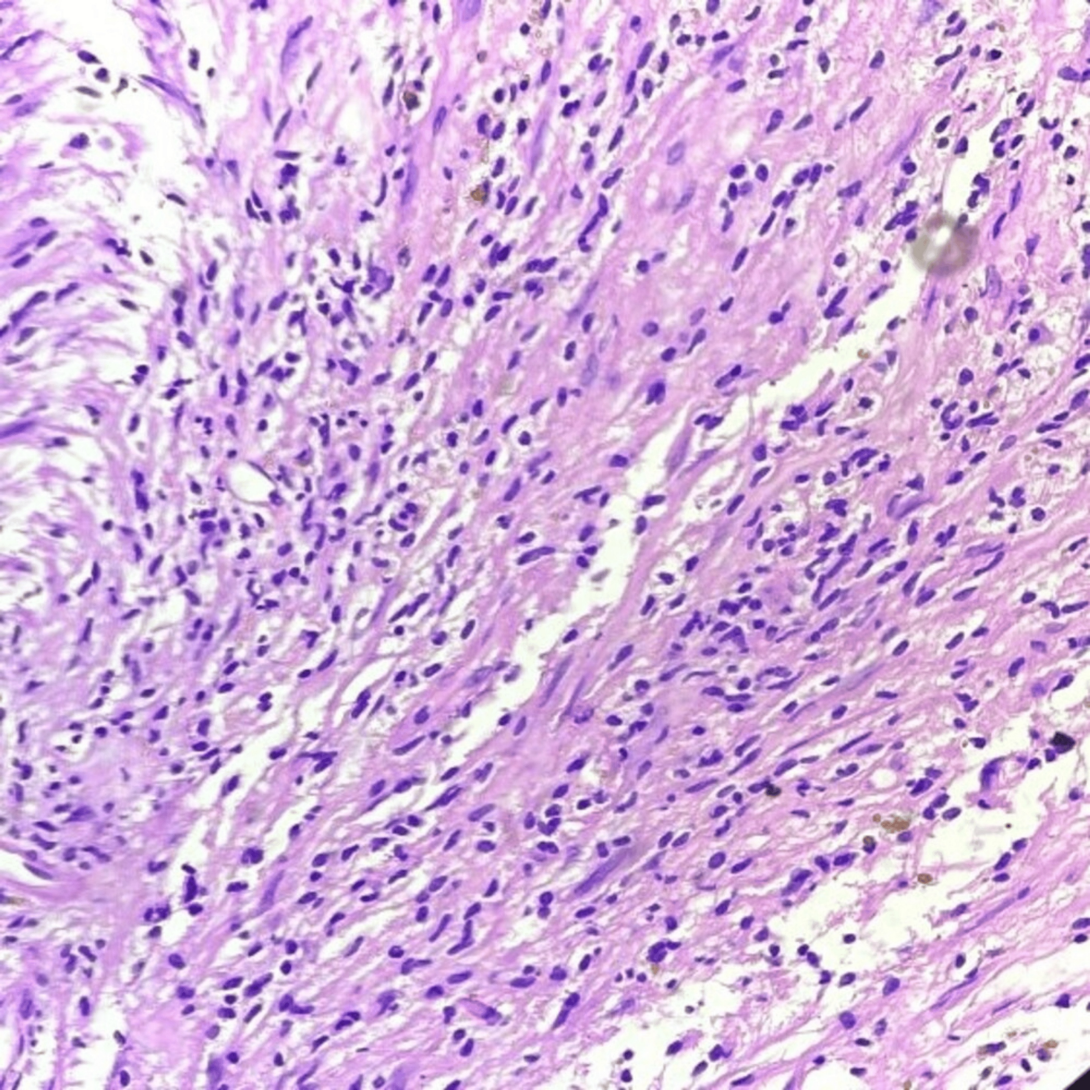
Photomicrograph (10x magnification) showing spindle cells arranged in fascicular arrangement with a surrounding population of lymphocytes and plasma cells on hematoxylin and eosin stain.

**Figure 3:**
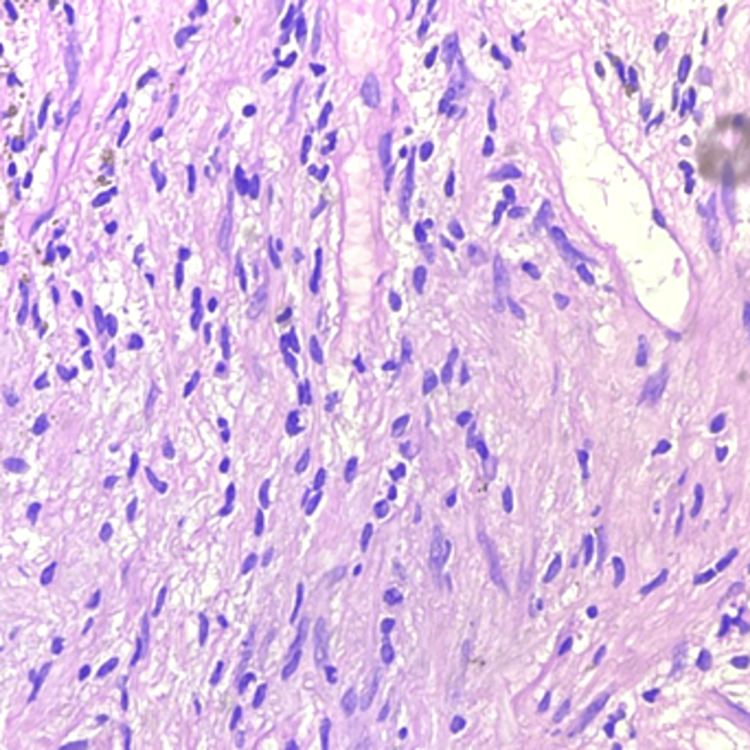
Photomicrograph (40x magnification) showing inflammatory cells with enlarged cells having spindle-shaped nuclei on hematoxylin and eosin stain.

A homogenous opacity across the entire upper lobe of the left lung was visible on the chest X-ray. An MRI was done to diagnose the lesion, revealing evidence of a well-defined hyperintense lesion, probably of neoplastic etiology (Figure 1). A biopsy was performed on the lesion at a private healthcare center. Biopsy reports were suggestive of adenocarcinoma of the lung. The patient reported to our cancer hospital for further treatment. After screening all her reports in a tumor board meeting, a decision to perform surgery was taken. A left pneumonectomy was performed as a last option for both therapeutic and diagnostic reasons. Grossly, the tumor was 7 x 3.2 x 2 cm in size, having a firm consistency. The tumor was grossly whitish and appeared homogeneous. Microscopy showed an increase in lymphocytes, plasma cells, eosinophils, and regular spindle cells arranged in fascicles (Figures 2, 3). A histopathological diagnosis of an IMT was given, and to confirm the diagnosis, immunohistochemistry was performed. ALK1 and SMA (smooth muscle actin) were detected positively by immunohistochemical investigation. Nevertheless, S100 had no effect on the tumor cells. These findings supported the diagnosis of an IMT. The patient was kept under medical observation, and antibiotics were administered for her recovery. The patient was administered IV Augmentin (1.5 g, TDS) and ceftriaxone (1 g, BD) for a duration of seven days. Postoperatively, the patient remained stable and was transferred to the ward. Follow-up was advised to the patient after 21 days.

## Discussion

According to the latest WHO categorization, IMTs are now classified as tumors with intermediate biological potential. The tendency for local recurrence and the negligible chance of distant metastasis are cited as the causes. The anatomical site determines the recurrence rate, which ranges from 2% for lung-confined tumors to 25% for extrapulmonary lesions. The spleen, lymph nodes, esophagus, stomach, salivary glands, breast, epididymis, central nervous system, and soft tissues are among the additional locations that have been documented [6,7].

There is variability and non-specificity in the clinical and radiological presentations. Consequently, unless surgical excision is carried out, it is challenging to establish the diagnosis [8]. Radiological data are non-specific and subject to variation. In 87% of instances, a mass or nodular lesion with uniform boundaries is found. Its diameter is between 1 and 6 cm. Typically, nodules are solitary but can occasionally evolve into multiples [3].

Vimentin and SMA reactivity was demonstrated by immunohistochemistry. In slightly more than half of the instances with cytoplasmic staining, immunohistochemical positivity for ALK is visible; it is less common near the nuclear membrane [9].

Histopathology generally shows a mixed spectrum of spindled cells arranged in fascicles and having myofibroblastic differentiation. Tumor cells exhibit a storiform pattern [10]. Differentiating IMT's compact spindle cell pattern from its many histological siblings is a laborious task. A noticeable inflammatory infiltration is seen in sporadic spindle cell sarcomas, spindle cell melanomas, and sarcomatoid carcinomas, which may only exhibit modest cytological atypia. In contrast, plasma cells typically do not constitute a significant portion of the inflammatory infiltrate in other forms of tumors.

The most common pattern observed has at least focally prominent nuclear hyperchromasia, atypical mitoses, necrosis, or vascular invasion, which are highly uncommon in IMT [11,12]. The histology traits listed above were consistently present in this case in addition to the IMT characteristics.

However, the outcome after complete resection is excellent; chemotherapy may be used for multifocal, invasive lesions. It can also be used in cases of local recurrence. Patients should be kept under observation and tracked at short intervals by bronchoscopy following the complete resection and thoracic tomography for any potential recurrence [3].

## Conclusions

Ultimately, the example of the lung's IMT highlights the intricacy and difficulties in diagnosing this uncommon disease. IMTs are benign but may be aggressive tumors, and a proper diagnosis frequently necessitates a high index of suspicion. A final diagnosis for our patient required a review of histological slides, radiological results, and clinical presentation. Surgery was the mainstay of the treatment plan, and it was successful in completely removing the tumor and reducing symptoms.

The significance of taking IMT into account when making a differential diagnosis for pulmonary masses is demonstrated by this instance, especially in patients who have non-specific respiratory symptoms. The importance of histology cannot be overstated, and the diagnosis can be further reinforced by immunohistochemistry staining, which identifies distinctive markers. Patients with pulmonary IMTs often have a good prognosis after complete surgical resection; nevertheless, ongoing surveillance is required to watch for metastasis or recurrence. The case study adds to the increasing corpus of knowledge on IMTs by highlighting the importance of interdisciplinary approaches and awareness in ensuring prompt diagnosis and treatment. Improving patient outcomes and deepening our knowledge of this uncommon ailment will benefit from additional studies into the pathophysiology and best management practices for IMTs.
